# Proximity and gaze influences facial temperature: a thermal infrared imaging study

**DOI:** 10.3389/fpsyg.2014.00845

**Published:** 2014-08-04

**Authors:** Stephanos Ioannou, Paul Morris, Hayley Mercer, Marc Baker, Vittorio Gallese, Vasudevi Reddy

**Affiliations:** ^1^Section of Human Physiology, Department of Neuroscience, Parma UniversityParma, Italy; ^2^Department of Psychology, Centre for Situated Action and Communication, University of PortsmouthPortsmouth, UK

**Keywords:** gaze, interpersonal distance, thermal imaging, autonomic nervous system, emotion regulation

## Abstract

Direct gaze and interpersonal proximity are known to lead to changes in psycho-physiology, behavior and brain function. We know little, however, about subtler facial reactions such as rise and fall in temperature, which may be sensitive to contextual effects and functional in social interactions. Using thermal infrared imaging cameras 18 female adult participants were filmed at two interpersonal distances (intimate and social) and two gaze conditions (averted and direct). The order of variation in distance was counterbalanced: half the participants experienced a female experimenter's gaze at the social distance first before the intimate distance (a socially “normal” order) and half experienced the intimate distance first and then the social distance (an odd social order). At both distances averted gaze always preceded direct gaze. We found strong correlations in thermal changes between six areas of the face (forehead, chin, cheeks, nose, maxilliary, and periorbital regions) for all experimental conditions and developed a composite measure of thermal shifts for all analyses. Interpersonal proximity led to a thermal rise, but only in the “normal” social order. Direct gaze, compared to averted gaze, led to a thermal increase at both distances with a stronger effect at intimate distance, in both orders of distance variation. Participants reported direct gaze as more intrusive than averted gaze, especially at the intimate distance. These results demonstrate the powerful effects of another person's gaze on psycho-physiological responses, even at a distance and independent of context.

## Introduction

The way that people communicate and engage in emotional and intentional exchanges needs the recognition of the subtle non-verbal cues that conspecifics generate (Freeth et al., 2013). Gaze (Frischen et al., 2007) and interpersonal distance (Baillenson et al., 2001) are important sources of social meaning, conveying a range of information regarding intentions (Nummenmaa and Calder, 2009), interpersonal relationships (Little, 1965; Evans and Howard, 1973), character (Argyle et al., 1974; Sodikoff et al., 1974), culture (Hall, 1966; Watson, 1970) as well as mental health and emotional state (Oliver et al., 2001; Aziraj and Ćeranić, 2013; Freeth et al., 2013). Competence in interpersonal interaction is important for reproduction and survival and therefore important from an evolutionary perspective. At the cognitive level this is achieved through the recognition of opportunities and threats whereas at the behavioral level, by the selection of social strategies for exploitation or avoidance (Bodenhauzen and Hugenberg, 2009). The autonomic nervous system (ANS) is an integral part of the social engagement process and alters its activity to foster behavioral strategies of threat engagement or non-emergency vegetative states (Porges, 2001).

Gaze, characterized as affording a “language of the eyes” (Frischen et al., 2007, p. 694), can communicate to the receiver a range of mental states, such as intentions, emotions, desires and beliefs. Emery (2000) states that gaze perception has evolved as a form of warning, informing the organism that a predator is attending to it. Many animals respond to being stared at with exhibitions of fear and submissive behavior showing that the identification of direct gaze is perceived as a warning (Schwab and Huber, 2006). Neuroimaging data have shown that the amygdala, a major structure for emotional processing, responds when individuals observe images of others engaging in direct gaze, rather than when they look somewhere else (Kawashima et al., 1999). Furthermore, the peripheral nervous system seems to be affected by gaze direction. Participants' skin conductance increases when observed by another, an indication that long periods of eye contact are perceived as threatening or aggressive (Nichols and Champness, 1971; Hoffman et al., 2007; Hietanen et al., 2008). People seem to be highly sensitive to being looked at by others showing finely tuned ability to detectothers' gaze (Baron-Cohen, 1995). Visual search experiments have shown that less time is taken to find eyes that are directed toward the observer than eyes that are looking at another target (Conty et al., 2006). Single cell recordings have shown that cells in the anterior part of the superior temporal sulcus code the social significance of the visual stimulus. Jellema et al. (2004) exposed rhesus macaques to a live 3-D live presentation of a human walking away or toward the subject in a both compatible (e.g., walking forward posture and head in the same direction) and incompatible manner (e.g., walking backward with head and body in the opposite direction). The researchers concluded that specialized cells in the temporal lobe analyze the intentions and goals of others actions. Moving from the normal population to clinical disorders, people with autism are reported to find eye contact aversive (Dalton et al., 2005). In fact compared to controls during tasks in which they are asked to explore the eye region of the face, people with autism show increased skin conductance as well as greater activity in the amygdala and fusiform gyrus. Their avoidance or dislike of eye contact suggests a strategy of physiological regulation (Dalton et al., 2005). Similar preferences and strategies are observed in people with social phobias (Horley et al., 2003).

The study of proxemics dates back more than four decades (Hall, 1959; Sommer, 1959). Hall (1966) defined interpersonal space in four different categories Intimate (0–46 cm), Personal (45–120 cm), Social (1.2–3.5 m), and Public (3.5 m+). Interpersonal space seems to be affected by a variety of individual and cultural differences (Hall, 1966). Women have smaller personal space when interacting with other women (Larsen and LeRoux, 1984) and, in contrast to males, dislike lateral intrusions into their personal space (Fisher and Byrne, 1975). Sanders (1978) showed that women maintain a larger personal space during menstruation. Moreover, in the United States, Malaysia, Spain, and Chile, irrespective of their country of origin and gender people preferred to be touched by a female rather than by a male (Willis and Rawdon, 1994). To some extent Spanish men were the most tolerant in terms of being touched by other males, whereas Malaysians irrespective of gender, were the least tolerant of being touched. Women from the United States had the highest tolerance in terms of being touched by the same gender. In the same line of research, Little (1968) asked Americans, Swedes, Greeks, Italians, and Scots to place a doll at the distance in which they would normally interact with another individual. Scots placed the doll at the greatest distance with Greeks placing the doll nearest. Age as well as prior knowledge about the forthcoming experience seems to also play a defining role in interpersonal distance. Older individuals prefer to sit further away from the interviewer regardless of expectations about the pleasantness or unpleasantness of the situation (Feroletto and Gounard, 1975). On the other hand younger individuals are affected mainly by their expectations about the situation. Perceived violence and level of criminality also affect personal space with people generally were less reluctant to sit next to an individual who has never committed a crime than to violent and non-violent offenders (Skorjanc, 1991). Studies with clinical populations have shown that schizophrenic patients, compared to controls, require larger personal space (Deus and Jokic-Begic, 2006) and people with anxiety, compared to individuals with psychotic disorders, left more space between themselves and the experimenter (Aziraj and Ćeranić, 2013).

Personal space seems to expand or shrink according the intimacy level of the participants According to equilibrium theory mutual gaze and personal space are two inversely related social behaviors (Argyle and Dean, 1965) modulated by intimacy: interpersonal space increases in order to balance out the effects of direct gaze. Interacting with avatars in a virtual environment, people leave more space between themselves and the virtual agent when direct gaze is involved; when the avatar invades their personal space, participants move further away (Bailenson et al., 2003). Data collected with electroencephalography and other psychophysiological measures is consistent with the above behavioral findings. When a male experimenter was looking at a male participant from a close distance, arousal was at its peak compared to when gaze was averted. In addition, when distance was increased arousal diminished; nevertheless, direct compared to averted gaze always caused greater arousal independent of distance (Gale et al., 1975). McBride et al. (1965) found that galvanic skin response (GSR) increased as a function of proximity and frontal confrontation. Similar results were observed by Nichols and Champness (1971). Finally, in a study that examined these effects in a clinical population, highly anxious women avoided gaze contact and exhibited backward head movements in response to male avatars who showed direct gaze. These behaviors were exhibited independent of distance (Wieser et al., 2010).

Porges' Polyvagal theory is one of the most influential interpretations on the role of the ANS in social engagement (Porges, 2001). Through evolution, the ANS retained three neural pathways whose hierarchy reflects their phylogenetic origins. On the top of this pyramid is the (a) social engagement system that is part of the parasympathetic nervous system (PNS) (Cannon, 1928), which inhibits more primitive structures of (b) mobilization (e.g., fight-or-flight), and (c) immobilization (e.g., feigning death, behavioral shutdown “syncope”). Having its main control component in the cortex, the social engagement system controls brainstem nuclei responsible for motor movements of communication such as eyelid opening, facial muscles, head turning, pharyngeal and laryngeal muscles, middle ear muscles, as well as muscles of mastication. These facial muscles have been reported to be dysfunctional in several types of psychopathology such as depression, autism, antisocial personality disorders and posttraumatic stress disorders. Despite head muscles, which are the “beacon” of human interaction, other structures such as the cardiopulmonary and the sympathetic-adrenal system alter their activity to support social demands and physiological relaxation. In occasions of threat the 10th cranial nerve (vagus) that controls the heart is disengaged providing an immediate cardiac output for mobilization of the organism without the need for activating the costly sympathetic adrenal-system. It is only after prolonged challenges that the sympathetic nervous system takes action. Mammalian evolution allowed the rise of this efficient mechanism that enables not only fast mobilization, but also fast physiological restoration as re-engagement of the vagal nerve inhibits sympathetic inputs to the heart (Vanhoutte and Levy, 1979). People with social or affective disorders do not show the same efficiency in dealing with environmental stressors as emotional arousal seems to engage lower, more primitive, structures associated with immobilization and energy saving rather than primary physiological responses such as heart mobilization and sympathetic engagement. Low cortisol reactivity has been linked to post-traumatic stress disorders (Yehuda et al., 1996), schizophrenia (Jansen et al., 2000), as well as to child neglect and abuse (De Bellis et al., 1994).

The majority of studies that have examined social attention and proximity were conducted decades ago with only a few managing to exercise full experimental control over the variables of interest. In fact to date, social attention research has largely been conducted in non-realistic experimental settings (Freeth et al., 2013) without the agent being physically present (Risko et al., 2012). Furthermore, only a few studies have addressed physiological elements of social arousal despite the fact that somatic arousal defines behavioral engagement strategies (Damasio, 1996). The current study aims to measure physiological responses in a more ecological experimental setting using high sensitivity functional thermal infrared imaging (fTII).

Thermal imaging is an upcoming physiological technique that allows recordings of cutaneous temperature variations wirelessly without interfering with the experimental procedure or the participant's biological movements (Pavlidis et al., 2012). Thermal imaging offers similar recording efficiency to GSR in reflecting autonomic effects in experimental procedures, without its problems of hitting ceiling levels of reaction (Kuraoka and Nakamura, 2011). Physiological observations of an affective nature are primarily related to subcutaneous vasoconstriction or vasodilation as well as heart rate and blood flow (Kistler et al., 1998). The validity of this technique for the measurement of various types of arousal has been demonstrated by simultaneous recording of proven measures such as GSR and Laser Doppler flowmetry (Kistler et al., 1998; Pavlidis et al., 2012).

The effects of social attention (direct gaze/averted-head-gaze) and proximity (social space—4 meters/intimate space—0.5 meters) on facial temperature are examined. The majority of studies measuring facial temperature have only looked at one site; in the current experiment multiple sites are examined in order to get a more accurate index of temperature fluctuations in the face as a function of condition. Temperature is measured from six regions of interest (ROI) on the face selected on the basis of previous research: (1) the nose (Nakayama et al., 2005; Kuraoka and Nakamura, 2011; Ioannou et al., 2013), (2) chin, (3) cheeks (Nakanishi and Imai-Matsumura, 2008), (4) periordital region (Pavlidis et al., 2001, 2002; Hahn et al., 2012), (5) maxillary area (Shastri et al., 2012), as well as the (6) forehead (Zhu et al., 2008). It is expected that the highest values of physiological arousal are going to be observed when the experimenter looks at the participant from intimate compared to social distance. In addition, being at a social distance will have a greater effect when the experimenter's gaze is directed toward the participant, rather than when averted. Finally, being at an intimate distance and not looking at the participant, will have a greater effect than when the experimenter is at a social distance, independent of gaze.

## Method

### Ethics

The Research Ethics Committee of the Faculty of Science of the University of Portsmouth gave approval for the study. Experimental procedures were in line with the declaration of Helsinki and the Code of Human Research Ethics of the British Psychological Society.

### Participants

Eighteen female participants were recruited for the study with an age range of 19–21 years old (*M* = 19.83, *SD* = 1.30). Exclusion criteria for participation in the study included gender (males), neurological or mental illness, as well as psychophysiological disorders. In order to improve the reliability of the physiological observation, consumption of vasoactive substances (nicotine, caffeine, alcohol) for at least 2–3 h prior of participation was prohibited. The female participants came from a range of cultural backgrounds and recruitment was performed through personal contacts and the University of Portsmouth recruitment database.

### Design

A 2 × 2 × 2 mixed factorial design was employed. The within subjects factors were gaze (direct gaze vs. averted gaze) and distance (social space vs. intimate space). The independent groups factor was order (intimate space then social space vs. social space then intimate space. The order of gaze vs. gaze aversion was fixed with the gaze aversion condition always occurring first. The dependent variable examined was face skin temperature on six sites on the face.

### Procedure

Upon arrival participants completed an Informed Consent Form, and then the BIS/BAS questionnaire (Carver and White, 1994). They were then escorted to the test laboratory where they were instructed to sit comfortably on a chair. Prior to the start of the experimental procedure, the participants were familiarized with a buzzer that was an integral part of the experimental protocol. During this period they were exposed to the sound of the buzzer, held the buzzer as it produced the sound and were fully informed about the reason why a buzzer would be used. Prior to any recordings the participants spent at least 10–15 min in the test laboratory. During the experimental procedure the female experimenter moved from intimate (0.5 m) to social space (4 m) or from social to intimate space. Visual floor markers were provided to the experimenter to define the precise distance from the participants, as well as other filler floor marks to avoid prediction of the experimental order. The transition from one experimental phase to another was signaled every 40 s by the buzzer. The buzzer sounded six times (a) the start of the experiment, (b) the second experimental phase, (c) the transition period from one social space to another, (d) the third experimental phase (e) the final phase as well as (f) the end of the experiment. Once the experiment was completed participants were given a self-report questionnaire regarding how uncomfortable or comfortable they found the four experimental conditions.

### Materials and data acquisition

#### Data acquisition

To perform recordings of skin temperature a digital Guide Infrared TP8 camera (ThermoPro™) was used with an uncooled FPA microbolometer (384 × 288 pixels). TP8 provides temperature sensitivity of 0.08 K with an accuracy of ±1°C and a sampling rate of 1 frame per second. Prior to recording the camera was placed 50 cm away from the participant, was automatically calibrated and manually fixated on the individual's face. The sampling rate was set at 50 Hz. The experimental room was set at normal temperature 20–21°C, 60–65% humidity, and with no direct sunlight, ventilation or airflow. Prior to the experimental procedure the participant were left for 15 min in the experimental room to acclimatize. All experimental recordings took place in the afternoon between 2–4 p.m. In addition behavioral recordings took place with a frame rate of 50 Hz, by two radio-controlled cameras, both connected to a DVD recorder The two video signals were combined using a Pinnacle system providing a two-split movie.

#### Questionnaires

To control for any personality variables that might have affected autonomic arousal (Critchley et al., 2001; Gaynor and Baird, 2007; Hughes et al., 2012) the BIS/BAS scale (Carver and White, 1994) was administered. For the current study a two-factor model of the BIS scale was used as suggested by Heym et al. (2008) where BIS is separated into BIS-anxiety, (4 items) related to conflicts, negative criticism etc. and the freeze/fight/flight system (FFFS-fear) that relates to fear responsiveness to punishment (3 items). The BAS scale is divided into three subscales (a) Drive for achieving goals (DR-4 items), (b) Fun-Seeking or Sensation seeking (FS-4 items, α = 0.73), and (c) Reward Responsiveness (RR-5 items). For the current study Chronbach's alpha value were for BIS-anxiety 0.57, for FFFS-fear 0.63, DR 0.71, FS 0.46, and RR 0.64. The relatively low Chronbach alpha values obtained may be due to the small number of items included each sub-scale. Nevertheless this psychometric scale is widely used, has good psychometric properties as well as good convergent and discriminant validity (Campbell-Sills et al., 2004). Furthermore four questions were given to the participants regarding pleasantness or unpleasantness of their experience. Rating were provided on a five point Likert-scale ranging from 1 = not uncomfortable to 5 = very uncomfortable. The questions were the following: (a) How uncomfortable/comfortable did you feel when the experimenter was looking at you from the back of the room? (b) How uncomfortable/comfortable did you feel when the experimenter was looking at you from a close distance? (c) How uncomfortable/comfortable did you feel when the experimenter was **not** looking at you from the back of the room? (d) How uncomfortable/comfortable did you feel when the experimenter was not looking at you from a close distance?

#### Thermal data analyses

Prior to the analyses the behavioral and thermal videos were synchronized in order to represent the same frame in time. Frames were extracted every 5 s using Launch GuideIR analyser by Wuhan Infrared Technology (http://www.guide-infrared.com). This was performed in a consistent manner across frames since participants' movements were minimal because of the nature of the experiment. For temperature extraction of the ROI different shapes were used as indicated by Ioannou et al. (2014). Circular shapes were used for the nasal tip, cheek, and the periorbital regions, whereas rectangular shapes for the maxillary area and forehead. Oval shapes were used only for the chin. The shapes did not vary in size across frames and temperature was extracted only when the face was in direct angle to the camera as it has been previously suggested that the above factors induce relative noise (Ioannou et al., 2013). On average 37 frames were extracted for each participant approximately nine for each phase. To perform the analyses the Statistical Package for the Social Sciences, version 17 (SPSS, Chicago, IL) was used. Data was screened to ensure it was suitable for parametric analysis. A reliability check was conducted. Results from five participants were analyzed by a second rater naïve to the purpose of the study. The second ratter performed temperature extraction on the same frames that were primarily selected for the five individuals (× 37 frames). In addition before moving into a Kappa measure of agreement the degree of temperature change from one condition to the other was calculated for both coders (see Table 1). Kappa's alphas (*p* < 0.05) ranged from moderate 0.64 (Cheek), to good 0.70 (Forehead, Periorbital), to very good agreement >0.8 (Nose, Chin, Maxillary). To eliminate the possibility that the results from the two ratters were different a 2 × 5 between groups' analyses of variance was conducted. No significant difference was observed between the two groups (*p* < 0.05).

**Table 1:** **The degree of temperature change from one condition to another based on the coding of two independent ratters**.

**NoseR1_R2**		**Maxil.R1_R2**		**Per.R1_R2**		**ChinR1_R2**		**For.R1_R2**		**Cheek.R1_R2**	
0.30	0.40	0.30	0.30	0.10	0.10	0.10	0.10	0.20	0.30	0.20	0.20
−0.50	−0.50	−0.10	−0.10	−0.10	−0.10	0.10	0.10	−0.10	−0.10	0.10	0.10
0.10	0.10	0.10	0.10	−0.10	−0.10	−0.10	−0.10	−0.80	−0.80	0.50	0.50
0.20	0.20	0.40	0.50	0.40	0.40	0.40	0.40	0.50	0.60	0.50	0.60
0.40	0.40	−0.70	−0.70	0.70	0.70	0.20	0.20	0.30	0.40	0.70	0.70
0.30	0.30	0.20	0.20	0.10	0.00	0.40	0.40	0.30	0.30	0.70	0.80
0.50	0.50	0.50	0.50	0.30	0.40	0.20	0.20	0.30	0.30	0.70	0.80
−0.10	−0.10	−0.10	0.00	0.80	0.80	0.30	0.30	0.60	0.50	−0.10	−0.10
−0.30	−0.30	−0.30	−0.30	0.80	0.90	−0.20	−0.20	−0.20	−0.20	−0.50	−0.50
1.00	1.00	0.80	0.80	−0.70	−0.70	0.30	0.30	0.60	0.60	0.40	0.40
−0.10	−0.10	0.40	0.40	0.30	0.30	0.10	0.10	0.20	0.20	0.20	0.30
−0.30	−0.40	−0.20	−0.20	−0.20	−0.20	0.40	0.40	−0.10	−0.10	−0.70	−0.80
0.00	0.00	−0.30	−0.30	0.90	1.30	0.00	0.00	−0.40	−0.40	0.30	0.30
0.00	0.00	−0.10	−0.10	0.00	0.00	0.00	0.00	−0.40	−0.40	0.00	0.00
0.50	0.60	0.20	0.20	0.00	0.00	0.50	0.50	0.10	0.10	0.30	0.30

## Results

### Correlations between temperatures on the six sites on the face

We correlated the temperature values from each site on the face with all other sites on the face for each condition. There were six sites on the face, which gives 15 correlations when all sites are correlated with all other sites. As there were four conditions this gives a total of sixty correlations. All of the sixty correlations were significant as were the means of the correlations for each condition (intimate space, gaze aversion *M* = 0.65, *SD* = 0.13; intimate space, gaze *M* = 0.67, *SD* = 0.13; social space, gaze aversion, *M* = 0.71, *SD* = 0.12; social space, gaze, *M* = 0.66, *SD* = 0.12). This is strong evidence that the different sites on the face are measuring a similar underling construct.

### Facial temperature analyses

**Figure 1:**
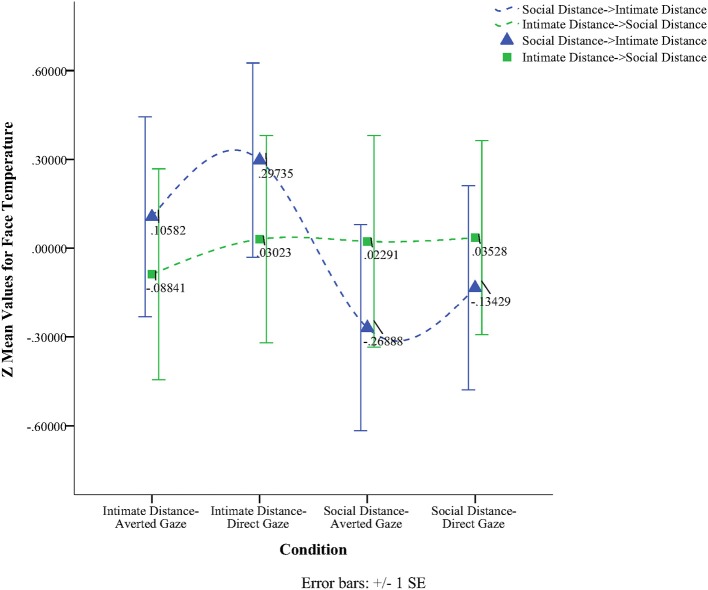
**Line graph illustrating the temperature of the face based on the experimental order of the four conditions**.

**Figure 2:**
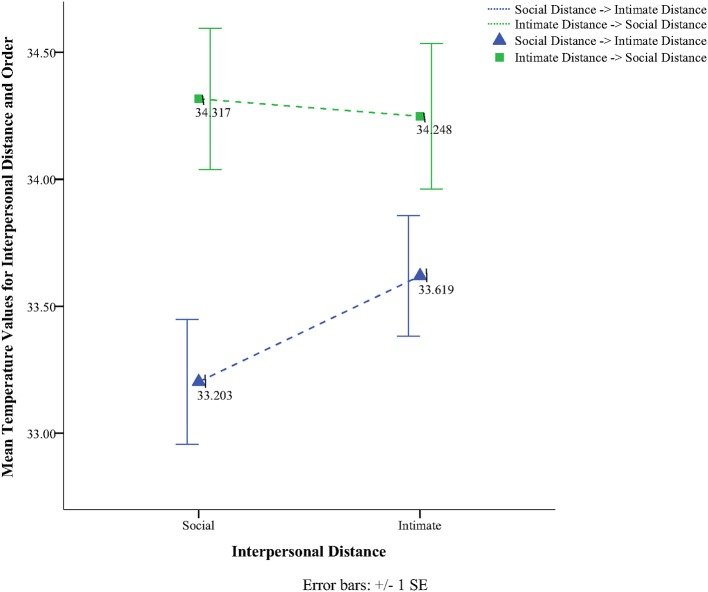
**Line graph representing the interaction effect between interpersonal distance and order**.

**Figure 3:**
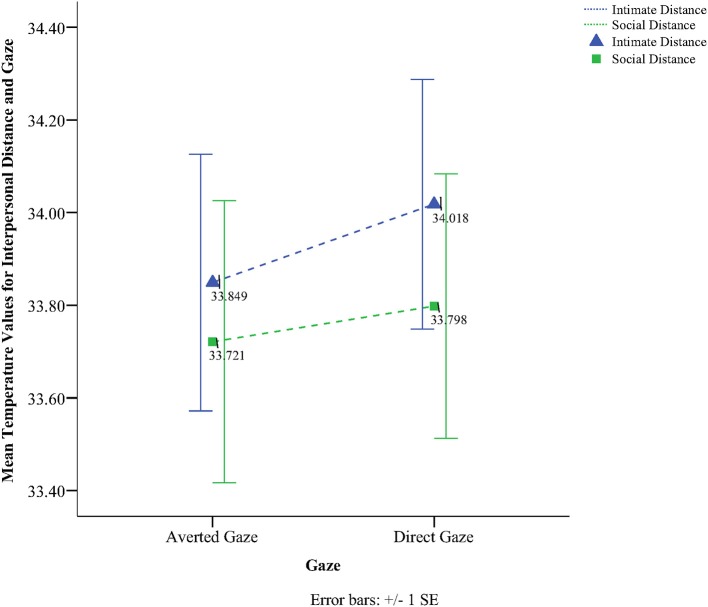
**Line graph representing the interaction effect between interpersonal distance and gaze**.

**Figure 4:**
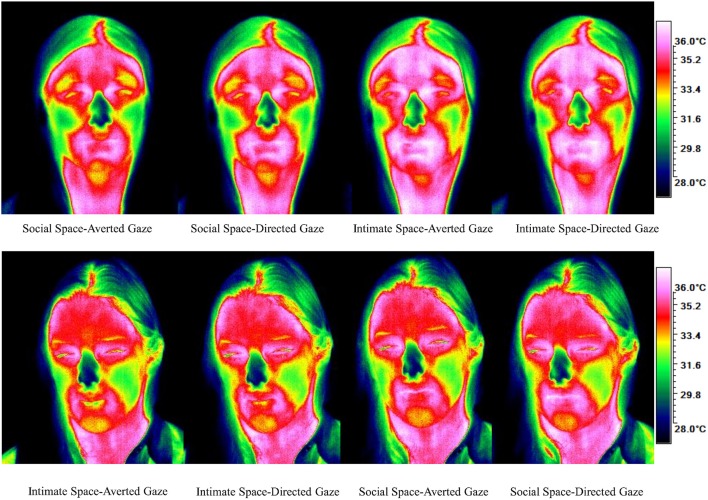
**Visual illustration of the development of temperature for each condition according to order**.

To obtain a more clear and robust pattern on the effects that interpersonal distance and gaze had on facial skin temperature, all ROI were averaged (see Table 2, Figure 1) and a 2 × 2 × 2 mixed factorial ANOVA was performed on the averaged data. No significant interaction effects were observed between interpersonal distance, gaze and order, Wilks' Lambda = 0.96, *F*_(1, 16)_ = 0.66, *p* = 0.429, η^2^_*p*_ = 0.04 or order and gaze, Wilks' Lambda = 0.80, *F*_(1, 16)_ = 3.89, *p* = 0.066, η^2^_*p*_ = 0.19. There was a significant interaction between interpersonal distance and order, Wilks' Lambda = 0.41, *F*_(1, 16)_ = 22.68, *p* = 0.000, η^2^_*p*_ = 0.58 (see Figure 2). From Figure 2 it seems that the interaction is a function of the fact that temperature increases when the experimenter moves from social space to intimate space but is relatively unaffected by distance when moving from intimate space to social space. This interpretation is supported by simple main effects analyses (with Sidak adjustment). It was observed that there was a significant increase in temperature when the experimenter moved from social space (*M* = 33.20, *SD* = 1.05) to intimate space (*M* = 33.62, *SD* = 1.01), *p* = 0.000. However, no significant difference was observed in temperature when the experimenter moved from intimate space (*M* = 34.25, *SD* = 1.22) to social space (*M* = 34.32, *SD* = 1.18), *p* = 0.054 (see also Table 3). There was also an interpersonal distance and gaze interaction, Wilks' Lambda = 0.76, *F*_(1, 16)_ = 5.03, *p* = 0.039, η^2^_*p*_ = 0.24 (Figure 3). From Figure 3 it appears that the interaction is the result of the fact that the effect of direct gaze increasing temperature is greater in the intimate space condition than the social space condition. Simple main effects analyses provide some limited supported for this interpretation as there was a significantly higher temperature in the intimate space, direct gaze (*M* = 34.02, *SD* = 1.14) condition compared to intimate space gaze aversion condition (*M* = 33.84, *SD* = 1.75), *p* = 0.000. The temperature was also significantly higher in the social space condition when the experimenter engaged in direct (*M* = 33.79, *SD* = 1.21) compared to averted gaze (*M* = 33.72, *SD* = 1.29) *p* = 0.014. However, the difference was greater in the intimate space condition (see also Table 3). There was a significant main effect of gaze with direct gaze having a higher temperature (*M* = 33.90, *SD* = 1.21) than the gaze aversion (*M* = 33.78, *SD* = 1.16), Wilks' Lambda = 0.36, *F*_(1, 16)_ = 28.35, *p* = 0.000, η^2^_*p*_ = 0.64, with a large effect size. Given the ordinal interaction between gaze and distance it is safe to interpret this main effect and conclude that direct gaze always produces a large effect on facial temperature. There was also a significant effect of interpersonal distance with temperature being higher in the intimate space condition (*M* = 33.93, *SD* = 1.15) than the social space condition (*M* = 33.76, *SD* = 1.23) Wilks' Lambda = 0.58, *F*_(1, 16)_ = 11.66, *p* = 0.004, η^2^_*p*_ = 0.42 with a large effect size. Given the interaction results we can again be relatively confident that there is a pervasive and robust elevation of temperature in intimate space. Finally in order to provide a visual illustration of the effects of the experimental protocol on facial temperature four images were created from two randomly selected individuals for each experimental order (Figure 4). The images were taken 10 s prior of the end of each phase. This was performed in order to allow enough time for large temperature effects to take place on the skin surface that would enable a vibrant visual illustration of the infrared image.

**Table 2:** **Mean Temperature values for the face according to order**.

**Region**	**Condition**	**Order**	***M***	***SD***	***N***
Face	Intimate space-averted	Intimate → Social	34.17	1.26	9
	gaze	Social → Intimate	33.52	1.04	9
		Total	33.84	1.17	18
	Intimate space-direct	Intimate → Social	34.31	1.24	9
	gaze	Social → Intimate	33.71	1.01	9
		Total	34.02	1.14	18
	Social space-averted	Intimate → Social	34.30	1.26	9
	gaze	Social → Intimate	33.13	1.07	9
		Total	33.72	1.29	18
	Social space-direct	Intimate → Social	34.32	1.16	9
	gaze	Social → Intimate	33.27	1.06	9
		Total	33.79	1.21	18

**Table 3:** **Results of the simple main effect analyses between distance and order as well as distance and gaze**.

		**Mean diff**.	***SE***	**Wilks' Lamda**	**F**	**Hypoth. df**.	**Err. df**.	**Sig. (two-tailed)**	**η^2^_*p*_**
Test 1	Intimate space (Order 1) vs. social space (Order 1)	0.417	0.072	0.324	33.44	1	16	0.000	0.676
	Intimate space (Order 2) vs. social space (Order 2)	−0.069	0.072	0.946	0.91	1	16	0.354	0.054
Test 2	Intimate space-eye contact vs. intimate space-averted gaze	0.169	0.034	0.389	25.10	1	16	0.000	0.611
	Social space-eye contact vs. social space-averted gaze	0.077	0.028	0.676	7.66	1	16	0.014	0.324

*Order 1, Social space → Intimate space; Order 2, Intimate space → Social space*.

### Individual region analyses

The mean values for each of the six ROI for each condition were calculated from all 18 individuals and mixed 2 × 2 × 2 mixed factorial ANOVAs were performed on the data for each region of interest. The repeated measures factors were (eye contact vs. head gaze aversion), proximity (social space—4 meters vs. intimate space—0.5 meters); and the independent groups factor was order (social space then intimate spaces vs. intimate space then social space). On all occasion the three-way interaction was not significant. However, two-way interaction effects between interpersonal distance and order were observed for the nose, cheek, chin, and maxillary area. In addition a significant interaction between distance and gaze was observed for the chin. In the “normal” order the pattern was very consistent. Temperature increased in all ROI when the experimenter moved from social space to intimate space. However, in the “odd” order the change in temperature was much less consistent. Main effects for distance and gaze were observed for the nose, maxillary area, cheeks, and the forehead. Temperature was higher in intimate rather than social space and higher in direct gaze compared to averted gaze. However, the effect of this latter could only be observed within the same interpersonal space (e.g., Intimate Space-Averted Gaze vs. Intimate Space-Direct Gaze). There was no significant main effect of order.

### Questionnaires

#### Questionnaire analyses

There was no significant correlation between BIS/BASS scores and temperatures on the six ROI. A One-Way ANOVA was conducted to examine the effect of the four experimental conditions (gaze aversion, intimate space, gaze aversion, social space, gaze intimate space, gaze social space) on the subjective pleasantness rating scores showed that unpleasantness scores on the four subjective questions were significantly different *F*_(3, 68)_ = 10.10, *p* = 0.000, η^2^_*p*_ = 0.3 with a medium effect size. *Post-hoc* comparisons using Tukey HSD indicated that unpleasantness scores were significantly higher when the experimenter was in intimate space and engaged in direct gaze (*M* = 4.17, *SD* = 1.1) compared to direct gaze in social space (*M* = 3.06, *SD* = 0.87), averted gaze in intimate space (*M* = 2.89, *SD* = 1.18) and averted gaze in social space category (*M* = 2.89, *SD* = 1.18). No other significant differences between groups were observed.

## Discussion

In the present study we explicitly modulated the social context in which gaze and proximity occurred by having two different experimental sequences. One sequence involved what would be considered a socially normal shift from a social distance to an intimate distance. The other involved a socially odd shift from an intimate distance to a social distance. At each distance there was a fixed sequence of the two gaze conditions: direct gaze always followed averted gaze. Analyses showed that when moving from social to intimate distance facial temperature rose on average by 0.42°C. However no significant temperature change between the two distances was observed when the socially odd sequence took place. On the other hand the effects of direct gaze compared to averted gaze were significant independent of order: direct gaze led to a higher temperature than averted gaze at both the intimate distance (a difference of 0.17°C) and at the social distance (a difference of 0.10°C). The subjective ratings given by the participants on the self-report unpleasantness scales supported the thermal findings: the highest “uncomfortable” scores were obtained by direct gaze in intimate and social distance followed by averted gaze in intimate and social distance.

Previous studies suggest that gaze and distance seemed to have a consistently robust effect on a range of psychophysiological measures. The current findings obtained using fTII are in agreement with results obtained with GSR (McBride et al., 1965; Nichols and Champness, 1971) and electroencephalography (Gale et al., 1975). As previously observed, direct gaze not only increases arousal but also seems to be mediated in intensity by interpersonal distance. Although in the current study the participants could not alter their interpersonal distance, the findings provide support for Argyle and Dean's (1965) equilibrium theory arguing that interpersonal distance and gaze interact to modulate arousal. In the present study, as interpersonal distance decreased, the arousal effects of direct gaze were greater. Overall it would appear that interpersonal distance and gaze interact to have strong effects on human physiology, with temperature variability of the face affected differently by each experimental condition. These bodily signs of autonomic arousal picked up by thermal imaging reveal the preparedness of the organism to support behavioral engagement strategies whether these involve social interaction or mechanisms of avoidance (Porges, 2001; Bodenhauzen and Hugenberg, 2009).

The increase in facial temperature creates a “physiological paradox.” Although the overall subjective experience of personal space intrusion as well as eye contact independent of distance was rated as uncomfortable, there was a rise rather than a dip in facial temperature. Previous literature using thermal imaging has found that negative emotions such as fear (Kistler et al., 1998; Nakayama et al., 2005; Kuraoka and Nakamura, 2011), stress (Pavlidis et al., 2012), and guilt (Ioannou et al., 2013), lead to a drop in the temperature of the nose, the maxillary area, the forehead, as well as the fingers as a result of peripheral vasoconstriction. From the present results it seems that the experience of interpersonal proximity and gaze does not fall physiologically into the same category. Increases in facial temperature have been observed in experiments of social contact (Hahn et al., 2012) and anxiety (Pavlidis et al., 2002; Tsiamyrtzis et al., 2007; Zhu et al., 2008). In the case of Hahn et al. (2012) participants were touched on various parts of the body by female and male experimenters using a handheld light-flashing device. Body parts that were touched were the face and chest (high-intimate) and the arm and palm (low intimate). What was observed was that when participants were touched on high intimate areas temperature increased, with an even greater increase when this act was performed by an experimenter of the opposite sex. These increases in temperature were localized on the nose, lip and peri-orbital regions of the face. Anxiety in individuals seems to cause a similar effect on facial temperature. Participants who were interrogated for a mock crime that they had just committed and who tried to defend their innocence showed an increase in temperature near the peri-orbital (Tsiamyrtzis et al., 2007) and the supraorbital vessels of the forehead (Pavlidis et al., 2002; Zhu et al., 2007). The results obtained by Pavlidis et al. (2002) are consistent with traditional polygraphs tests that use physiological measures of pulse, blood pressure, perspiration and skin conductivity to draw conclusion about the honesty of the individual. Behaviorally, something that is common in the above experiments is a challenging social situation. In extent although physiologically, evidence exist explaining the reason why this might have happened in the case of Pavlidis et al. (2002), Zhu et al. (2007) as well as Tsiamyrtzis et al. (2007) no evidence exists to explain why this might have been caused in the study by Hahn et al. (2012). Pavlidis and Levine (2002) suggested that temperature increase results from increased blood perfusion to the surface of the skin. Increased blood perfusion is the result of increased delivery of blood to body tissue and the heart is the organ of the body that can sustain such a function (Kreibig, 2010). Thus judging from the previous literature on thermal imaging, increased blood flow to the surface of the skin is the result of increased heart rate that causes the skin temperature to rise. The current physiological findings as well as the observation made by previous research are in support of Polyvagal theory (Porges, 2001). According to this theory mammalian evolution favored the development of an efficient neural control model, which provided increased control of the heart via the myelinated vagus, the 10th cranial nerve. When needed, sympathetic tone expression and increase cardiac output supports transitory mobilization states without activating the costly sympathetic or adrenal system; only if vagal disengagement can provide safety from short-lived stressors. Furthermore, the findings of the current study are not agreement with Bell et al. (1996) as the proposed “stress theory” should also have the appropriate temperature tendency. In the current set-up a rise in temperature was observed instead of a drop (Kistler et al., 1998; Pavlidis et al., 2012; Ioannou et al., 2013).

No significant order effect was observed, however, in the “odd” sequence what both Figures 1, 4 have in common is that no significant temperature change took place from one condition to the other and only minor temperature changes can be observed. Approaching the individual initially from intimate distance and then moving to social space yielded no significant temperature changes. Although at the group level, significant results were obtained in direct gaze compared to averted gaze and independent of sequence, this outcome might have reached statistical significance because of the power of the normal experimental order (Social distance → Intimate distance). The way that the experiment was designed seems to favor the approach moving from social distance to intimate distance as a linear increase in temperature was observed. In this order physiological effects seem to intensify from one condition to the other. On the other hand, moving from intimate to social distance, temperature changes did not behave in the same manner. What can be observed by the “odd” experimental sequence is an overcompensation effect where temperatures started off higher and decreased less. Although in intimate distance, an increase in temperature from averted to direct gaze was observed, no temperature change took place during the transition from intimate to social distance. Facial temperature did not have the opportunity in a 40 s time interval to reject physiological changes that took place at intimate distance along with direct gaze before moving to social distance and averted gaze. It is believed that these results show a physiological temperature “spill over” effect from the most arousing condition to the next as can be seen during the transition from intimate to social space.

Literature on thermal imaging does not provide evidence on the time needed for facial temperature to return back to baseline or rest values when the temperature of the face rises. However, temporal evidence exist on how temperature behaves when temperature decreases. Nakayama et al. (2005) reported that after stimulation, 220–280 s are needed in order for temperature of the nose to return to baseline values. Moreover Kuraoka and Nakamura (2011) reported that changes on the nose lasted on average for 60 s before descending back to baseline values. Evidence from rodents suggests that according to the region of interest there is also the appropriate expected delay (Vianna and Carrive, 2005). The back, head and the body of the animal took approximately 60–75 min to return to baseline whereas the eyes, tails and paws 14, 10, and 15 min consecutively. Although changes in heart rate take place much more rapidly than changes in vasoconstriction (Kistler et al., 1998; Vianna and Carrive, 2005) and despite the fact that the two physiological phenomena have different underlying mechanisms we believe that there was not enough time in the present study in the transition from intimate to social distance for adequate heat changes to be observed between conditions. Finally although temperature changes in the direction of the physiological excitation can be rapidly observed within 15–20 s (Kistler et al., 1998; Nakayama et al., 2005; Kuraoka and Nakamura, 2011), as a result of an affective stimulus temperature restoration takes substantially longer.

The current experiment demonstrates that this novel, wireless, physiological technique has the sensitivity not only for picking up changes in the intensity of the stimulus but also in replicating results that have been observed by previous studies using other widely established physiological measures. Through this experimental model the foundation stone has been set for other studies in the clinical domain whether this relates to diagnoses or the efficacy of treatment (De Bellis et al., 1994; Yehuda et al., 1996; Jansen et al., 2000; Horley et al., 2003; Dalton et al., 2005). Thermal imaging is a valuable tool in studies in which participants cannot express their emotions verbally (Nakanishi and Imai-Matsumura, 2008; Kyselo and Di Paolo, 2013; Uithol and Paulus, 2013) or in studies where emotional arousal can only be inferred by coding behavior and by measuring physiological responses (Vianna and Carrive, 2005).

Functional thermal imaging has the potential for identifying subtle psychophysiological changes that take place on the surface of the skin as a result of underlying vasoconstriction or heart rate variability. Although in the current experiment a rise in temperature was observed no direct other physiological measures were obtained to clearly explain why such changes took place. Literature on the topic provides some evidence as to why this might have happened and we can only speculate that this is related to increased heart rate output based on the findings from the literature (Pavlidis and Levine, 2002). It would be important in future studies that investigate temperature changes to employ heart measures to explain temperature related physiological observations. In addition related to the context of the current study are the other two distances “personal” and “public” which would also be nice to investigate (Hall, 1966). Furthermore since in the current experiment female participants were exposed to female experimenters, mixed gender dyads could be added as well as mixed gender groups. Some of the temperature changes observed might have resulted from the reflection of heat from the experimenter. However, given the strong psychophysiological effect of the experimental conditions and orders, this is unlikely to account for the changes measured Future research could attempt to measure and exclude such effects. Finally, thermal imaging has a poor temporal latency despite its sensitivity in picking up small fluctuations in temperature. This is not because of the inadequacy of the technique but because of the temporal latency that the skin needs to exhibit changes of physiological nature whether these are the results of vascular constriction or of increased blood flow. Thus it is important that in experiments that do not follow a linear increase in the intensity of the presented stimuli to leave adequate time from one condition to the next in order for temperature to return to approximately baseline values.

## Conclusions

Interpersonal distance and perceived gaze are two related social constructs with each one imposing its presence on the physiological reactions of the receiver. Current observations of these phenomena suggest that direct compared to averted gaze affect autonomic reactions and facial temperature. These results persist independent of the distance from which gaze occurred. In terms of interpersonal distance, intruding an individual's intimate space led to a marked increase in temperature. This result however was only evident when there was an approach from social to intimate distance. On the other hand a difference in temperature was not observed when the individual was approached primarily by the experimenter in intimate distance and then moved to social distance. This phenomenon of “physiological spill-over” represents an effect that lasted longer than the pre-defined time interval of the experimental phase. Skin temperature did not recover after it was exposed to the most arousing intimate condition and this effect lasted after transition was made to the least arousing condition in social space. Despite the methodological significance of the study, gaze and interpersonal distance have their own piece of the pie to claim in social interaction. Physiological reactions obtained by facial skin temperature suggest that preparatory action for engagement or avoidance takes place by the organism when gaze is engaged and when intimate space is violated. However at the level of conspecifics and as suggested by the physiological reactions of the participants, social elements of space and gaze are not treated as threatening since, if they were, a drop in temperature showing the full blown effects of threat would have been observed. These results suggest rather, a physiologically preparatory action by the organism for what will follow whether this is an attack or a pleasant social interaction.

### Conflict of interest statement

The authors declare that the research was conducted in the absence of any commercial or financial relationships that could be construed as a potential conflict of interest.
